# Functional Neurologic Disorders, disorders to be managed by neurologists, or are neurologists wandering in a dangerous field with inadequate resources?

**DOI:** 10.3389/fpsyt.2023.1120981

**Published:** 2023-03-17

**Authors:** Marco Onofrj, Paola Ajdinaj, Anna Digiovanni, Naveed Malek, Giovanni Martinotti, Filippo Maria Ferro, Mirella Russo, Astrid Thomas, Stefano Luca Sensi

**Affiliations:** ^1^Department of Neuroscience, Imaging, and Clinical Sciences, “G. D'Annunzio University” of Chieti-Pescara, Chieti, Italy; ^2^Center for Advanced Studies and Technology (CAST), “G. D'Annunzio University” of Chieti-Pescara, Chieti, Italy; ^3^Barking, Havering, and Redbridge University Hospitals NHS Trust, London, United Kingdom; ^4^Department of Clinical, Pharmaceutical and Biological Sciences, University of Hertfordshire, Hertfordshire, United Kingdom

**Keywords:** somatic symptom and related disorders, factitious disorder, utilization behavior, deception, hysteria

## Abstract

In recent years, some neurologists reconsidered their approach to Medically Unexplained Symptoms and proposed Functional Neurologic Disorders (FND) as a new entity, claiming that neurology could offer alternative treatment options to the psychotherapies provided in psychiatry settings. FNDs, for this purpose, should include only the disorders listed as Conversion from the Somatic Symptom and Related Disorders (SSRD) group. The present review analyzes the rationale of this position and challenges the arguments provided for its support. The review also discusses the systematization of these disorders as provided by public health systems. It outlines risks stemming from economic support and public funding uncertainty, given their negligible epidemiological dimensions resulting from the parcellation of SSRD. The review underlines the unresolved issue of Factitious Disorders, which are in the same SSRD category of the international classification but are, nonetheless, overlooked by the theoretical proponents of the FND entity. Comorbidity with other psychiatric disorders is also analyzed. We propose a model that supports the continuum between different SSRD conditions, including Factitious Disorders. The model is based on the emergence of feigned death reflex and deception from frontal lobe dysfunction. Finally, the paper summarizes the wealth of historical psychiatric and psychodynamic approaches and critical reviews. The study also puts in context the categorization and interpretation efforts provided by the most eminent researchers of the past century.

## Introduction

Functional neurologic disorders (FND) came back into the clinical repertoire of neurologists only in the last 20 years, after decades of neglect and misconceptions, according to editorials and position papers published in several Neurology journals (1–9). A new scientific society, the Functional Neurological Disorders Society (FNDS), was founded in 2017 to improve the quality of care for FND patients. Several widely high-impact scientific papers have been published in the last two decades. These publications addressed the issues of the identification of positive features (1, 2), and social stigma linked to previously used definitions like “Hysteria” or “psychogenic” or “psychosomatic” disorders, referring to FND (3, 4). They also focused on treatment opportunities offered by physiotherapy (5, 6), as well as speculated by pathophysiology theories mainly centered on the representations of the body, movement, and volition (agency) rather than on psychodynamic mechanisms (7).

Editorials and commentaries in various neurology journals highlighted the impact of FND (and Somatic Symptoms Disorders) on neurological practice, a phenomenon so pervasive that some authors described it at “epidemic” levels (8, 9).

The 2013 edition of the Diagnostic and Statistical Manual 5^th^ edition (DSM-5) (10) accepted the term FND as an equivalent to *Conversion disorder.* FND were also placed inside the category of Somatic Symptom and Related Disorders (SSRD), together with somatic symptom disorder-Briquet Syndrome (SSD), Illness anxiety disorder-hypochondria, psychological symptoms occurring during other medical conditions, and Factitious Disorders (Table 1). The DSM-5 (10) also describes, for each subcategory, epidemiological and associated features, culture-related factors, comorbidity, and prognosis, providing essential clues on procedures and discussions that were the background for the classification (duly prefaced, as for other DSM versions, by the explanation that DSM is a guide to shared nomenclature, not an easy alternative to training in psychiatry).

**Table 1:** Somatic symptom and related disorders—adapted from DSM-5.

Somatic Symptom Disorder
Illness Anxiety Disorder
Conversion Disorder (Functional Neurological Symptom Disorder)
Psychological Factors Affecting Other Medical Conditions
Factitious Disorder
Other Specified Somatic Symptom and Related Disorder
Unspecified Somatic Symptom and Related Disorder

**Figure 1:**
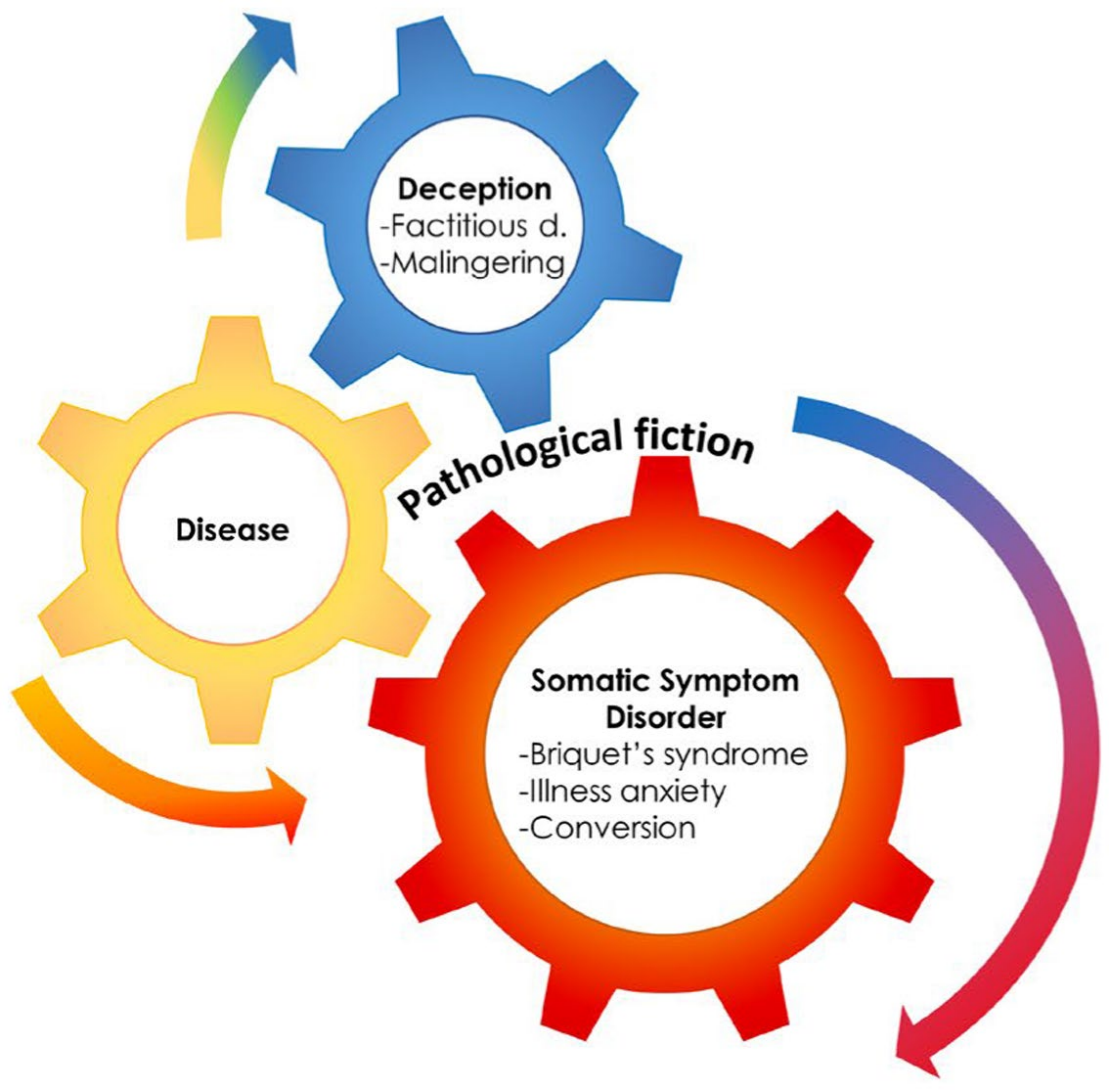
The presence of a medical condition can often mesh with deception and somatization, supporting the hypothesis of an hystero-malingering continuum. Reproduced, with modifications, from Feldman and Yates, 2018.

Theoretical models and laboratory evidence of abnormal network connectivity and neurotransmitter unbalance in FND patients helped conceptualize FND as a unique disorder, separable from the somatoform (11–13) and somatic symptom (10) disorder categories, even if it has been placed in the category of SSRD. This framing represents, however, a challenge to the conceptualization of FND as a unique entity, as DSM-5 implies the five entities of SSRD to be within the spectrum of FND. Moreover, the DSM-5 highlighted associations with other psychiatric disorders, with the most challenging concept being the inclusion of Factitious Disorder inside the category. Factitious Disorders are based on the deception enacted by the patient, that is, the pretense of having a medical or psychiatric disorder with the patient genuinely unaware of enacting such deception (14). This is truly a major challenge, as it introduces concepts of self-deception in SSD and the disavowal of intention (15). Some authors, coalescing in 2001 to present a book on contemporary approaches to Hysteria, proposed a continuum encompassing Hysteria, Conversion, and Factitious Disorder, as reformulated in recent publications (16–18) (Figure 1).

Our paper addresses the topic of FND and SSD in detail. It also calls to action to encourage a multidisciplinary approach that bridges the gap between the disciplines of neurology and psychiatry. The paper also underlines the need to reconsider the seminal psychopathological approach (19).

We also summarize the long-standing history of psychodynamic interpretations of FND, frame it into the broader category of SSRD, analyze the unresolved issue of Factitious Disorders as well as Malingering, discuss the evidence for various treatment options, and suggest a model for the disorder.

## The arguments in favor of a neurologic reclassification of FND

The prevalence of patients with FND or Medically Unexplained Symptoms (MUS, including fibromyalgia, chronic fatigue syndrome, and reflex sympathetic algodystrophy/causalgia) in neurology clinics is estimated to be around 16–30% (2). Several authors have described such a high prevalence as representing a crisis, or a silent epidemic (8), invoking calls for newer approaches to reaching out to help patients with FND. As an extreme example of the attitude, an authoritative editorial wrote that “the patients do not want to hear that they have a psychiatric disorder and they go from doctor to doctor, psychiatrists do not seem interested anyway, and the prognosis is terrible” (19), and the concept was plainly reported in a very recent paper (20).

Several reviews and position papers (1, 21) have detailed the main reasons to separate FND into a category that can cover a well-demarcated group of patients acceptable in much wider neurology practice settings including out-patient and in-patient settings. Citing evidence and views of the authors from these papers (1, 21), the main reasons included the following issues:

1. The pattern of motor presentations was consistent through time. Foot dragging, knee buckling, give-away loss of muscle tone, arm fall without pronation, or distractible motor disorders like dystonia, tremor, or myoclonus, which are the most common motor presentations of FND in patients accessing neurology clinics in the last decade (2), are the same features in patients previously dubbed as hysterics in scientific reports from the early 20^th^ century (22). The above-mentioned features, together with the variants of Hoover’s sign (23), could be considered examples of “positive” features for the diagnosis of FND, as opposed to diagnoses of FND based on the exclusion of medical causes.
2. Treatments based on physiotherapy provide better results than psychotherapy (24). Earlier studies suggested that a trial of physiotherapy was better suited for patients than psychotherapy, as providing a “saving face” solution (18). More recent studies have focused on the possible effect of motor retraining (25–27) yet were less dismissive of psychotherapeutic or other approaches.
3. Several studies have underlined that FND, if untreated, persists for a longer time during follow-up (28, 29). A few years ago, a systematic review showed that up to 40% of patients with FND report similar or worse outcomes at 7-year follow-ups (28).
4. Some studies using imaging techniques in FND showed hypoactivation of the contralateral primary motor cortex, decreased activity in the parietal lobe, aberrant activation of the amygdala, increased temporo-parietal junction activity, and hyperactivation of insular regions. Functional connectivity shows aberrant connections between the amygdala and motor areas, temporoparietal junctions, and the insula (7, 30, 31). The unintentional, unconscious, production of motor or sensory symptoms was framed into models of top-down processing of sensory experiences, recapitulating the concept of “priors” i.e., the unconscious memory of prior experiences predicting the outcome of the present experience (32, 33). The models suggest that a distortion of the preparatory motor output is the mechanism of motor FND (30), akin to the hypothesized model of dystonia (31) and the model mechanism of its treatment with botulinum toxin (34, 35).
5. Stressful life events during childhood or adulthood are often quoted among the predisposing or precipitating factors for FND (36).
6. Overlaps between FND and neurological disorders like Parkinson’s disease (37–42), Dementia with Lewy bodies (37, 38, 42), epilepsy (43), or multiple sclerosis (44) have been previously described, showing that FND could precede or accompany the onset of these conditions (45, 46).
7. In several discussions, the model underlines the absolute absence of access to consciousness of FND symptoms (15, 47–49), thereby suggesting a difference from Factitious Disorders and Malingering, the latter two being generated by deception, thus a supposedly volitional act (50). Several reviews quote the DSM-5 categorization (10), in which factitious disorders are placed in a separate category of somatic symptoms disorders (which, nonetheless, include FND). Many discussions on FND compare this disorder to Malingering (16, 17), overlooking or ignoring the complex question posed by the imposing presence of Factitious Disorders.

### Counterarguments and objections

**Figure 2:**
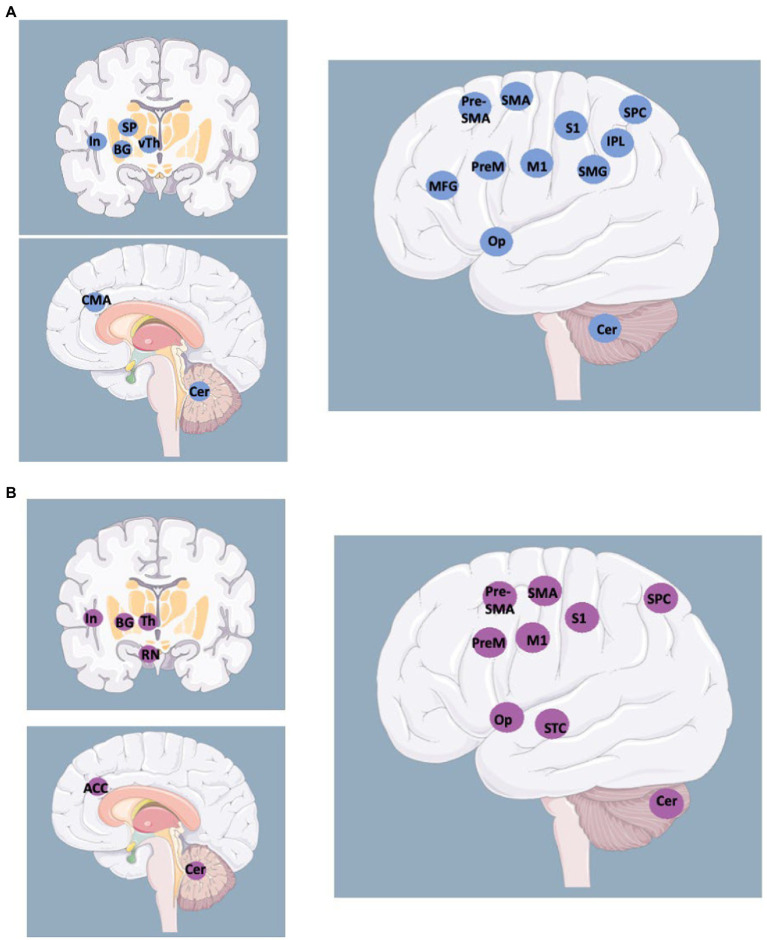
Activated areas during voluntary movements revealed by fMRI studies **(A)** vs. activated areas during involuntary movements revealed by fMRI studies in patients with alien limb and other movement disorders **(B)**. BG: Basal Ganglia; Cer: Cerebellum; CMA: Cingulate Motor Area; In: Insula; IPL: Inferior Parietal Lobe; M1 primary motor cortex; MFG: Middle Frontal Gyrus; Op: Operculum; PreM: Premotor cortex; RN: Red Nucleus; S1: primary sensory cortex; (pre-)SMA: Supplementary Motor Area; SMG: SupraMarginal Gyrus; SP: Striato-Pallidal complex; SPC: Superior Parietal Cortex; STC: Superior Temporal Cortex; (v)Th: (ventral) Thalamus. http://smart.servier.com.

1. The phenomenological descriptions of various motor FND may be simply due to physical limitations rather than represent a pattern of a specific disorder. For instance, due to the mechanical limitations of muscle-joint ranges of motion, patterns like foot-dragging or knee buckling seem the only options available to enact a loss of function of muscles around a joint in a limb. Moreover, specific patterns are only seen in some *ethno-psychiatric* disorders, i.e., disorders present only in some geographical areas affect people of some ethnic backgrounds. For example, the sensation of shrinking of the penis, termed Koro (51, 52), is only described in Asian and African populations, occasionally with epidemic presentations. The explosive bursts of violence or aggressive agitation are only described with the Amok of South-East Asia (52, 53). The hyper-motor choreic and myoclonic manifestations of the Jumping Frenchman of Maine (54) or Latah (52, 55), were geographically and temporally limited, in episodes that were finally interpreted as Mass Hysteria (53, 56), same as the dancing disease that hit Strasbourg (57) at the end of the 16^th^ century (58). These examples are evidence of a culturally influenced manifestation of conversion/FND symptoms in limited geographical areas. Recent puzzling evidence of cultural influence was provided by the epidemy of TikTok Tourette, which found room on social and general media (59, 60).
2. Reports of the efficacy of physiotherapy in FND patients suffer from the same selection biases burdening many studies on the effect of physiotherapy and other therapies organized in complex settings, i.e., need for sufficiently blinded study designs (5). The number of selected patients is often small, matching of controls is absent, and cross-overs or inferiority designs have never been attempted. There are no studies comparing psychotherapy with physiotherapy on the long-term outcomes in FND nor with other techniques claimed to be efficacious in FND, like hypnosis, transcranial direct current or magnetic stimulation, mindfulness training, narrative exposure treatment, or approaches through alternative medicine (61–63).
3. The long-term persistence of FND symptoms is probably biased by patients’ cohort selection. Cases presenting late with other severe diseases, medical or neurologic, do show that Conversion, fibromyalgia, and reflex sympathetic dystrophy may disappear once that new clinical entity supersedes (7, 40, 64). The only paper describing follow-up functional Magnetic Resonance Imaging (fMRI) assessments of FND shows a reduction of symptoms (65). In contrast to recent position papers on FND (66), prior DSM versions (11, 13) reported that Conversion is time-limited. That FND and SSD could be time-limited conditions was also underlined by several psychiatric and psychoanalytic studies (16, 18), as shown by the puzzling question posed at the early times marked by claims on end or crisis of psychoanalysis, i.e., “where did all the hysterics end up?” (16, 18). A preposterous example related to where the hysterics disappeared can be found in the investigations on this disappearance. After the seminal demonstrations by Charcot and colleagues on the phenomenology of Hysteria (67), based on the presentation of the affected patient to the audience, the topic faded from general attention. However, some curious researchers investigated the follow-up of the patients who had served as eminent examples during the seminars in the clinic. Not surprisingly, some of the demonstrative cases were later found to be still available for practical demonstrations of Hysteria, given the provision of a modest fee (68).
4. The idea that Conversion may be determined by an underlying brain disorder dates back to the first descriptions of the condition. Charcot believed that eredo-degeneration was the subtending mechanism (67, 69), Freud was aligned with the hypothesis by his mentor (70, 71), several psychiatrists re-proposed the concept (72, 73), by suggesting explanations that were based on neurophysiological knowledge of the time, i.e., cortical hyperexcitability and enhanced response to reticular system afferents was the hypothesized mechanism when the reticular system was the ultimate discovery of neurosciences (74). Studies on fMRI activation of cortical or subcortical areas during voluntary or involuntary movements (movement analysis protocols) are scarce (30, 75–77), which is in contrast with the many studies on resting state connectivity (77). Any resting state (fMRI) connectivity study can only provide inference on putative networks, as it does not record what happens during a motor behavior but only shows the set of networks statistically evidenced during rest. With task studies performed during a voluntary movement, fMRI shows activation of multiple areas, including the motor cortex, the premotor cortex, primary and secondary sensory cortices, supplementary motor area and contralateral cerebellum, invariably also the frontal lobes, parietal lobes, and temporal lobes in the majority of volunteers for these studies (Figure 2). Activations of the same areas were observed during a triggered movement in a patient with putative involuntary movements of the alien hand, anterior type, and in 36 patients affected by Tourette syndrome, during the putatively involuntary but suppressible tics (35, 78, 79). Moreover, there are no studies on involuntary movement prototypes, like chorea, ballism, rubral tremors, and L-dopa-induced dyskinesias. This is because the studies are technically complex, there is no back-averaging program software for fMRI, and the time resolution of fMRI is limited to 0.5 s or more, far more than the time resolution of electrophysiology and the time of reflex circuit activations, thus making it impossible to catch the temporal sequence of recruited areas. Therefore, the variability of voluntary activations during movements performed upon control conditions makes the results of studies in putative, involuntary movements less than contentious. Moreover, recent reviews had to admit powerful limitations, “many different techniques, tasks, and heterogeneous clinical samples were used, rendering any attempt to do a meta-analysis difficult. Most studies had several limitations, among which small sample size and confounding factors were the most frequent” (80). As a concluding criticism, we must also underline that in the few fMRI studies attempting a comparison with FND patients, the matched group was made of healthy controls instructed to feign a paresis (81–83). This selection introduces the relevant bias constituted by access to consciousness of the aberrant motor behavior, with the consequential question about what the difference is showing. Are we seeing differences in the network subtending the motor act or in allowing or impeding access to consciousness? If the latter is likely, how would the disavowal, i.e., the psychopathological denial, of access to consciousness, and its effect on volitional networks, be accounted for? Not secondary, all SSD, including FND, are burdened by frequent association with other psychiatric disorders, as discussed in the next section. Accurate matching procedures should provide control groups burdened by the same or similar comorbid patterns (personality disorders, obsessive–compulsive disorders, or even patients affected by SSD who did not show Conversion/FND features). With this matching, the comparison would rest on more solid grounds and provide an observation on the different effects of suggestibility, which was considered the core feature of Hysteria by Babinski (84, 85).
5. The frequency of trauma and stressful life events was only modestly different between patients with FND and controls or patients with hand dystonia (86). But the hypothesis on childhood trauma as the predisposing etiology of FND is challenged by the century-old observations, which were the seminal findings of psychoanalysis. Sigmund Freud only in his early case descriptions, identified traumatic experiences (i.e., sexual harassment during childhood) as the predisposing or precipitating factor for conversion disorders (87). However, shortly after his first few studies, when he felt challenged by the inconsistency of recalled histories, he developed his seminal theories on the fantasy of trauma, interpreted as a psychodynamic mechanism structured unconsciously in defense and denial of the intrapsychic conflict of ideas and affects (87). Several earlier studies on Hysteria, Conversion, Somatoform and Factitious disorders did not find any significant prevalence of trauma in early life (18). In psychotherapy, a technique recently emerged, the Eye Movement Desensitization and Reprocessing (EMDR), which seemed to be specifically suited for the treatment of trauma (88), but no studies of its effect on FND/SSD have been attempted.
6. FND appear frequently in patients with parkinsonism (up to 59%) before or after the onset of motor symptoms (89), and pseudo-seizures are frequently coexistent with epileptic disorders (43). FND overlaps, or pseudo-relapses, are a consistent problem in Multiple Sclerosis management (44). Studies in patients with parkinsonism have uniformly shown that FND can predict the occurrence of cognitive decline and coexist, often, with hallucinations (89, 90). Often FND remit when dopaminergic treatments are initiated but recur when cognitive decline supersedes (40). However, these studies were based on a different hypothesis rather than simply providing evidence that FND may be the expression of a brain disorder. That evidence had already been provided by studies on post-encephalitic parkinsonism (91–93). It was not casually coincident with the birth of psychodynamic theories. The purpose of the studies on parkinsonism was to understand whether the development of FND was linked to the same dysfunction which leads to the occurrence of hallucinations. In other terms, to conceptualize FND as an expression of the weakening of frontoparietal control networks and subsequent loss of consensual reality and emergence, disinhibition, of the internal narrative generator, the posterior cingulate gyrus (89, 90, 94). It might be argued that FND appearing in the presence of cognitive decline and psychosis are different from FND appearing in the absence of both. It is also debated whether the findings in neurologic disorders should constitute the model, to be dissected in “formes frustes,” in the quest for understanding mechanisms and treatment. With these comparisons, it will be possible to elucidate whether FND/SSD are a form of psychosis rather than a separate disorder.
7. Nevertheless, the main flaw of the explanatory theory relies in the simplified demarcating line separating unconscious from willed behaviors and considering Factitious Disorders as akin to Malingering (37), which is not a factitious disorder, and was, in some studies, used as a comparator for FND. Malingering is in fact not a medical term and is not listed as a diagnosis in DSM-5 (10). In malingering, the motivation (gain) is external such as receiving money (95) (Figure 1).

## The main criticism: The unsolved and overlooked problem of factitious disorder

Factitious Disorder obtains a definite identity only in DSM-III (11). Therefore related interpretations are likely biased by the prior insufficient separation from other SSD and from Malingering. Before DSM-III, Factitious Disorder and malingering were considered primarily present in the military (in conscripted subjects) and the criminal world (96). For some authors, a catalyst of Factitious Disorders and Malingering was the creation of social welfare states with access to financial compensations or unnecessary care (18).

In the DSM-IV (12), the Factitious Disorder was also termed “Munchausen Syndrome,” according to the early presentation of the disorder by R. Asher (1951) (97), but DSM-5 disfavors its use. The reasons for opting for other terms are discussed in detail by Feldman and Yates, 2018 (98), where attention is focused on the forensic categorization of the disorder and the need to highlight the abusive behavior in legal terms.

The core definitions of Factitious Disorders are that these “are conditions in which a patient intentionally produces or feigns physical or psychological symptoms…without obvious secondary gain” (ICD-10 definition) (14). DSM-5 states that “the motivation for the behavior is to assume the sick role” but, despite stating that deception and feigning are the core element allowing a diagnosis, it also says that “assessment of conscious intention is unreliable” and that the chance to conclude for a diagnosis is linked to the chance to incur into evidence of feigning (10). Therefore, guidelines (10) state that deception and the absence of external incentives for the behavior are diagnostic criteria, yet they mention that the intention of feigning cannot be reliably assessed. Any assessment should be capable of identifying deception and consciousness of deceiving by separating the elements of voluntary activity. However, assessments of deception and feigning are far from being accomplished, far from a resolutive operating technique, and surrendered to legal, judicial, rather than medical matters. Because pathological lying (pseudologia fantastica) is a critical component of factitious illness, it is argued that the clinician should actively seek its identification. Pathological lying is distinguished from “normal” lying by several characteristics, including the recurrent, enduring, and compulsive presentations, the fantastic, self-aggrandizing content, the possible ego-dystonic structure with maladaptive or destructive outcomes for the quality of life of the person involved (98).

Psychodynamic interpretations describe in primary gain (i.e., the solution of an intrapsychic conflict) the origin of Factitious Disorder, at difference with secondary gains, which are the practical or economic benefits resulting from the enactment of a distinct behavior (1). Primary gain was described as keeping an internal conflict or need out of awareness, and secondary gain as avoiding a particular activity that is noxious and getting support from the environment that otherwise might not be forthcoming. However, the primary gain is also the origin of Conversion, to the point that several studies depict a continuum from Conversion to factitious to malingering (98) (Figure 1).

Psychodynamic studies highlight that “factitious disorders are famously difficult to treat medically and are highly refractory to psychotherapy” (36). Only a few reports could describe the interaction between a therapist and a patient affected by Factitious Disorder. Many authors resorted to writing that communication was burdened by deception and opposition. Some authors (99) describe confrontational management once that deception was documented. Many authors underline the oppositive defiant response to attempts to rationalize and explain the behavior, constantly leading to the concluding words “what if that’s true?,” a comment underlining the scarce access to consciousness of the behavior.

The most interesting report from a psychotherapy session was from one of the few collaborative patients, who was describing herself as “desperate to try and get help”: she wrote “I despise myself for all the things I have done and have continually tried to stop what is like an addiction.” Similar, descriptions can be found in the recent book by Feldman and Yates (98).

The unconscious origin of the feigning behavior is interpreted as a drive to be in control of medical conditions which were previously experienced as painful, i.e., that these disorders are repetitive compulsions motivated by the desire for mastery (e.g., taking forced control over the medical personnel providing care) (36). Feigning, or inflicting damages to a proxy in a state of blurred consciousness, is therefore interpreted as the expression of an unconscious wish to enact a personal drama and to reinforce the strength of a relationship with medical professionals who figure in the fantasy lives of those affected by the disorder (100). In the 1978 draft of the DSM-III (11), the motive subtending Factitious Disorders was described as the *compulsion to act out a sadomasochistic relationship with physicians regarded as parental figures.*

Most psychodynamic interpretations were produced before DSM-III set a category for Factitious Disorders, and close reevaluation of the described cases often unveils a mixture of SSRD (Conversion or FND) and factitious disorders in the same patients (101–104).

## Psychiatric and psychodynamic approaches

### Classifications and medical disciplines involved in FND management

The International Classification of Diseases (ICD), in its ICD-10 version (14), classified FND only under the category of psychiatric disorders, and the ICD-11 version (101) listed only functional tremor, functional parkinsonism, and pseudo-seizures among disorders classified as neurologic disorders.

Most national health services do not list FND among their Diagnosis Related Groups (DRG) (102), which benefit from cost coverages for hospitalizations from neurology clinics. Among the European nations, only the United Kingdom (UK) National Health Service (NHS) considers reimbursements to neurologists *via* their NHS Trusts under the coding category of “neuropsychiatric disorders.” It must also be underlined that, in UK NHS, there is a peculiar disproportion, as compared with other countries, between the number of psychiatrists and neurologists. The ratio is 1 to 8, at difference from Italy, where ratios are 2 to 1. Further differences must also be underlined in the different targets of the two disciplines in different countries. In UK and Germany, psychiatrists manage dementia cases, which are dealt with by neurologists in other countries. It is, therefore, possible that the urge to frame the category of FND into neurology was dependent on the contingent organization of UK’s NHS, while in other countries, the discipline of psychiatry has far wider access to long-term management, social access, and approaches to legal issues than the discipline of neurology.

The national health care systems of countries providing free health care or even insurance-based health care are commonly based on coding or the DRG (102) assessment of disorders to evaluate whether access to health care can be economically supported and justified. The deployment of DRG coverages varies remarkably among countries, as indicated previously. In the USA, the Insurance based coverage is subject to posttreatment screening, with the risk of direct charges to the patient.

In Italy (102), Switzerland, France, Belgium, the Netherlands, and Germany, all DRG related to Conversion/FND and including the category of somatoform/somatic symptoms disorders are recognized only for the discipline of psychiatry.

As a final comment, it should be underlined that, recently, several editorials and analyses appeared in the US scientific literature, underlining the need for better psychiatric training for the residents in neurology. The studies highlighted the inadequate training of neurologists and their troubles when dealing with conditions overlapping with psychiatry, suggesting several projects to improve knowledge, as opposed to the current 4 weeks-training (103).

### Comorbidity or associations

The association with anxiety and depressive disorders as well as the association of one SSRD with the others, is quoted for all categories. While the association with obsessive–compulsive disorder is uniquely quoted for illness anxiety disorder, the association with dissociative disorders is quoted in DSM-5 (10) for conversion/FND, and in DSM-IV-TR with histrionic, antisocial, borderline, and dependent personality Disorders. The association between Factitious Disorder and borderline personality disorder is quoted in DSM-IV-TR (13). Factitious Disorder was removed from the DSM-IV (12) category “Dissociative Disorders” (i.e., multiple personality disorder, dissociative amnesia) to the DSM-5’s SSRD group. Factitious Disorders may appear in association with other mental disorders. DSM-5 (10) quotes the association between the other four SSRD and dissociative disorders. DSM IV-TR (13) included histrionic, antisocial, borderline, and dependent personality disorders. DSM-5 (10) is not shying away from defining that “some aspects of factitious disorders might represent criminal behavior.”

Adoption studies of somatization disorders (100, 104–106), analyzing the occurrence of somatization in adopted patients affected by somatization disorders, and personality traits of natural parents, showed clearly that somatization disorder was associated with heritable personality traits such as a predisposition to antisocial behavior and substance abuse (18).

## Interpretations of FND and SSD

When investigating Hysteria and teaching his conclusions to Freud, Charcot explained that hysteric symptoms were the expression of the loss of “function” and were associated with hereditary neurodegeneration. This was to pursue the disclosure of the “physical” background of disorders (18, 67, 107, 108).

Babinski offered an edge-cutting definition of Hysteria, by writing that “Hysteria is a disorder caused by suggestion, treatable by persuasion” (84, 85, 109), but this definition did not survive, as the underlying optimism was eventually challenged by a massive amount of evidence.

Also, Freud (18) was aiming at a “physical” explanation of disorders and framed his explanations within the confines of the dominant scientific knowledge of the time, thus energy, entropy, and displacement of energy were the pillars of his metapsychology.

Several studies of the second half of the last century (18), hypothesized that conversion disorders could be different from other psychosomatic symptoms, and linked to cortical hyperexcitability and insufficient inhibition of afferents from the reticular system, the structure which was the ultimate discovery at the time (107, 108).

The organic, physical, mechanistic hypotheses thus appear embedded in the history of neuropsychiatry, the use of the term “functional” is far from new, and references to the dominant knowledge-based theories of the time are recurrent, as in all other aspects of scientific endeavor (Kuhn, Feyerabend) (110, 111).

With the development of psychoanalysis and psychodynamic theories, the interpretation of somatic symptoms disorders was, however, mostly ascribed to mental functioning, defense mechanisms, and coping styles (18, 67).

After his early, retracted, description of trauma as a predisposing factor, Freud (18) reconsidered his interpretation and developed his ideas of Conversion as the representation of the unconscious attempt to compromise between drive (pulsions) and repression (defense mechanisms). Freud identified as “primary gain” the result of this unconscious coping strategy, i.e., gaining advantageous stability against the emergence of a hostile drive and the psychic cost of denying its existence. He identified, subsequently, “secondary gains” in the relational advantages (including economic gains) which could be derived from the enacted behavior (87).

This seminal theory is still the bedrock of psychodynamic interpretations of the expression of mental disorders: defense mechanisms are analyzed in DSM versions and coded as narcissistic, immature, neurotic, and mature to explain the underlying psychodynamic mechanisms of mental disorders. Accordingly, conversion symptoms and hypochondriasis are listed among the immature defense mechanisms (112).

The interpretation of the mechanisms of somatic symptoms disorders has been multifaceted, hedging on different, often divergent theories produced by psychoanalytic schools (18).

Jung ascribed Hysteria to intense, “exaggerated,” manifestations of the conflict with proximal (relational or familiar) figures, introducing in this veiled interpretation, the concept of hostile attitude and production of the somatic symptom to deny and hide hostility (113). Jung agrees with Freud that hysterical symptoms are the return of repressed memories in the patient’s personal background.

This concept reemerges in several psychodynamic studies on somatization (18), going as far as suggesting that hypochondria underlines an evil personality and that somatic symptoms are produced to constrain the unconscious emergence of hostile attitudes, or that somatic symptoms are produced as an atonement of hostile feelings to obtain the primary gain, with denial, and a secondary gain by playing the sick role, thus obtaining a legitimate way to get dependency needs met (112).

A similar interpretation by K. Leonhard suggested that “hysteria has a purpose,” contrary to hypochondria, which is the expression of angst.

The nosology effort made by K. Leonhard [114–117]deserves further attention. This author, leading, at his times, the neuropsychiatric services of the Berlin Charité Hospital, third heir to the psychiatry school of Wernicke, and Kleist and to the gigantic German psychiatry schools (114, 115) of Kraepelin (116, 117), Bleuler (118) and others, produced outstanding disease classification systems, which were often anticipating concepts that reappeared only lately in literature and classification systems.

K. Leonhard described Hysteria (119–122), with other SSD, as a symptom of different mental disorders rather than considering it a disease. This concept, with which we agree, anticipates the DSM-IV TR classification methods, based on multiple axes of disease categorization, which highlights the coexistence of multiple expressions of mental disorders. The multiaxial categorization was abandoned by the DSM-5 manual, which was instead attempting core definitions of the different disorders.

Janet agreed with Freud that fixed subconscious ideas were at the core of Conversion and suggested that “a narrowing of the field of consciousness” was responsible for symptoms, linking somatic symptom disorders to dissociative disorders (18).

Several psychoanalysts (18) continued to interpret Hysteria along the original Freudian lines, in terms of psychosexual fixations, referring back to pre-genital stages of development (i.e., anal or oral) (123–125). Others shifted the paradigm in terms of object fixations as the drivers of the primary gain mechanisms (126–128).

Lacan (129, 130) produced new theories focusing on the fragmented infantile experience of the ego (mirror stage), which is painfully constructed through experience, and on re-emergence of fragmentation in psychosis, of which he attempted to decipher the language. His studies led to further development of psychoanalysis as a theory of meaning and communication, rather than causal sequences. Hysteria was interpreted as the paradigm of the symptom as a symbol or metaphor of unconscious needs or drives. Lacan described the fragmented experience of the body as the original self-representation, which evolves through comparisons in the mirror stage, to form an integrated body experience when separating from the mother’s body.

Hysteria in Lacan’s writings is one of the four “Discourses” constituting the essential models of communication between a self and the other (130). The four “discourses” are Master, University, Analyst, and Hysteria, representing the modalities of communication, addressed to a personification of the agent (an unexpected precursor of the recent emphasis on agency), to knowledge, to search for truth, and to the attempt to reach the “other.” In this theory, Hysteria is not just the product of imaginary anatomy. It is a main structure of communication, and several Lacanian psychoanalysts, as Lacan himself, use the term hysteric to highlight a modality of communication attempting to build a social bond with the other, i.e., Hysteria is not simply the somatic symptom but a modality of communication attempting to link the “other” to questions posed by the self. In some over-simplifications Lacan writes that the introduction of Hysteria as a structure of communication can lead to the paradoxical conclusion that “we are all hysterics.” Beyond the structural placement, many definitions of Hysteria in Lacanian literature are outstanding: Hysteria as a communication “which cannot be mastered by knowledge and therefore remains outside of history,” Hysteria as imago of the fragmented body, “hysteria is a chimaera, bringing to the mind the myth of the sphinx,” i.e., acting as the sphinx posing a riddle to man, where the riddle is a recurrent request for recognition of identity through the answer of the questioned man; the hysterics start out with “I am what you say” and end with “all of what I am you cannot say” i.e., the hysteric is asking for recognition, but the recognition is always insufficient and prompts a renewed question.

Later studies focused on general models of personality (18), including dimensions of temperament and character, structured in a temperament and character Inventory, which provided strong reliability, regardless of cultural background, gender, or age. The four dimensions of temperament (131) are listed as harm avoidance (serotoninergic), novelty seeking, reward dependency (dopaminergic), and persistence, and the three dimensions of character (131) are listed as self-directedness, cooperative and self-transcendent. Studies of patients with conversion and somatization disorders, conducted based on the personality assessment, revealed a link with the multidimensional structure of personality, consisting of high harm avoidance and high novelty seeking, with low self-directedness, and commented on the frequent association with borderline and avoidant or obsessive personality disorders. These personality studies (131, 132) underlined the multidimensional background of patients with somatic symptom disorders, suggesting that understanding the prevalent traits would be crucial for treatment planning and management, including the choice of psychotropic drugs and psychotherapy techniques (133, 134).

In philosophy, then in psychology, three main elements of voluntary (willed) actions (135) were considered as possibly representing separate processes: (a) volition, or will, defined as the power that the mind has to order the forbearing of an idea; (b) agency, as the perception of being the one who chooses or enacts the action; (c) intention, as a conscious choice of who is the agent of the action. For example, in organic weakness or paresis, there is intention, volition, and agency of the effort to perform an act but no motor effect; in utilization and imitation behaviors of frontal lobe diseases there is no intention nor volition, while agency, i.e., perception of being the one who performs the act, is preserved (although possibly blurred by the frontal lobe lesions that induce the stimulus bound behaviors); in sensory deafferentation, or posterior alien hand symptom, there is no agency, i.e., the moving arm is not perceived as part of the body.

In FND/Conversion, the definition of the missing elements is less clear; originally the focus was on volition, “they say, I cannot; it looks like I will not; but it is I cannot will” (136), therefore FND was interpreted as dependent on disordered will. The implication of conscious or unconscious mechanisms, however, blurred the definition. The psychodynamic model invokes an unconscious mechanism acting independently of consciousness. Thus a disordered unconscious intention might give rise to a disordered will. From these hypotheses, an unconscious intention to action would rely on a simultaneous (conscious) intention to act. Lacanian interpretations (129, 130) of FND talked of “failed acts” either as identification with an external will or as an act within a fantasized body space. Attention to the motor act, as part of intention, was then called into a mechanistic cause, also to explain the distractibility of FND. It must be pointed out that when invoking attention into a mechanistic cause, a paradox surfaces, as the maintenance of unconsciously derived FND symptoms requires conscious attention (2, 137) as shown by the fact that FND are, by definition, distractible by entrainment maneuvers.

More recently, the focus (1, 32, 33) has been on agency, which was considered as the expression of disordered perception, altered by insufficient suppression of the “priors” recruited by top-down processing of perception (32, 33, 138). The interpretation would be that there is impaired access to consciousness of the somatic symptom in FND.

In patients with Factitious Disorders, the agency of feigning and perhaps volition of feigning are denied and canceled from consciousness; intention should be consequently unconscious.

Further attempts at interpretation with psychodynamic analyses focused on self-deception, avowal, and disavowal of action, suggesting that disavowal of action (18), which becomes not intended, is acted to avoid disturbing the patient’s image of his/herself (18, 48, 49). The concept of avowal did not separate FND from feigning, and, unsurprisingly, the dissection of volition led to a discussion of moral contents, distinguishing an unconscious, morally neutral deception, where it is the self and not the other, whom the deceiver is deceiving; this is different from a deception being a willed act, which is pure malingering.

The boundaries between the various discussed disorders were therefore considered unclear and a possible continuum was suggested, and the “Hystero-malingering continuum” appears in the literature (10, 16, 17, 48, 67, 98). For example, it was shown that hysteric patients misinterpret evidence and selectively attend to only a certain part of the overall evidence. These patients who employ such strategies as positive and negative misinterpretations, selective attending, and selective evidence gathering were considered motivationally biased (primary, secondary/economic gain), and can therefore be self-deceived (48).

## Psychotherapeutic treatments

A throughout review of 2017 (139) analyzed the different treatment options for SSD/FND.

FND management and treatment strategies include: (1) patients education, (2) psychotherapy, (3) psychopharmacological treatment, and (4) physical rehabilitation, with basic (and essential) avoidance of medical harm (66). Thorough communication is required to enhance the chances of successful treatment. The patients must be informed about the nature of their disturbances (140) and the existence of a “real” problem must be acknowledged with the patient, as well as its possible reversibility with appropriate treatments (25), to improve compliance with therapies and the final outcomes. Subsequently, the therapy should be tailored to the patient’s necessities and clinical features, which can be assessed by the Psychosomatic Evaluation (PE) (139). PE encompasses the patient’s intrinsic features, such as the personality and the presence of a Type A behavior (prone to competition, irritability, and psychomotor urgency), as well as the occurrence of external stressors that modify the allostatic burden causing an acute or chronic overload that ultimately impairs wellbeing (139). The absence of adequate social support should also be assessed, as it is related to worse outcomes (141). The complex ties between “external” and “internal” proneness to somatization underlie the production of one or more maladaptive disease behaviors, like health anxiety, hypochondria, thanatophobia, or disease phobia, or – on the opposite side – illness denial (139). Other peculiar manifestations include the “anniversary reaction” and, finally, FND or persistent somatizations (139). The PE may be particularly suitable to monitor the therapy response (139), while psychotherapy and pharmacological therapy may be effective for FND treatment and relieve concomitant psychiatric disorders. The psychodynamic approach has been successfully tested (142), both in acute and chronic settings (143). One of the main limitations of the psychodynamic approach is the relatively slow effect compared to other techniques. However, short-term psychodynamic psychotherapy (STPP) is also effective in treating FND, as confirmed by a meta-analysis, and may represent a promising therapeutical resource (144). Cognitive Behavioral Therapy (CBT) is now widely employed (145–147). CBT shows both early and long-term efficacy (148) and is effective in the reduction of PNES (149, 150). A cognitive-behavioral intervention, namely Treatment of Anxiety and Physical Symptom (TAPS), has been successfully applied to a cohort of pediatric and adolescent patients with persistent somatic complaints (151). Other techniques have been tested in FND patients, with different degrees of success (e.g., mindfulness, stress management, hypnosis add-on, prolonged exposure therapy, and group psychoeducation (152–157)). Antidepressant and concomitant anxiolytic treatment may also be helpful (158).

In the review, physical therapy is also analyzed and considered as generally well-tolerated and effective for motor symptoms (66, 159), alone or as part of other multidisciplinary approaches, considering that it can also include psychotherapeutic interventions. Symptom remission may be sustained up to one year after cessation (66, 159–161). However, physiotherapy strategy should be planned according to guidelines and to the patient’s profile to retain adherence and achieve the best results (25, 160), explaining to patients the interpretations of their physical symptoms to help them with the management of their situation and physical condition. In this way, a powerful working alliance can be built between physiotherapists and patients, which can improve the patients’ quality of life.

The authors also summarized the effect of Transcranial Magnetic Stimulation (TMS), which can improve the clinical picture, according to several observations (162–164), but they also commented that the mechanism is still unclear, addressing methodological bias (165). Interestingly, TMS was suggested to mainly act through a cognitive-behavioral mechanism (166), rather than by cortical excitability modulation. According to this model, TMS-related improvement of FND would result from suggestion and from motor re-learning (i.e., the assessment of the capacity of normal movements), as training for self-agency during involuntary movements. Finally, antidepressant and concomitant anxiolytic treatments were analyzed (23) and benzodiazepines for catatonia (167).

Other studies discuss using Narrative Exposure Treatment (NET), classical Hypnosis, Trauma Desensitization (52). Hypnosis has been studied in two randomized clinical trials in patients with FND, including symptoms of sensory loss or speech disturbance showing an improvement comparable to psychotherapy (168, 169). It can be used also to demonstrate the diagnosis of FND to patients if the symptoms disappear under hypnosis. NET is usually used as a treatment for trauma disorders, particularly in individuals suffering from complex and multiple trauma (170–172). The idea behind it is that people who write or tell their own stories can also change the biological mechanisms that are at the origin of the trauma (52) because the systematic narration of their life can have a consolidating effect on how one perceives itself, as Bures writes in his “Geography of madness” (52, 173).

## Other (pharmacological) treatments

Current therapeutic options for FND are mainly non-pharmacological. Although antipsychotic drugs.

have no direct indication for FND treatment, they are prescribed off-label in over 60% of cases (174). According to a national survey (174), olanzapine (42.1%), quetiapine (15.8%), and risperidone (5.3%) are the most prescribed antipsychotics for SSD treatment. Other atypical agents (like brexipiprazole, aripiprazole (175), and paliperidone (176)) are also effective as an add-on (177). These findings align with the model that considers FND and SSD as psychotic symptoms (90) arising from a dysfunctional coupling of task-negative and task-positive networks, jointly to an aberrant Salient Network activity (42) and a weakened frontal control system (178, 179). In line with this model, MRI spectroscopy (180) in SSD patients revealed the presence of high Glx (glutamate+glutamine) levels within the posterior cingulate cortex. These levels were higher than healthy age-matched control subjects and correlated with catastrophizing pain symptoms. From a speculative point of view, anti-glutamatergic agents can counteract the hyperactivation of the Default Mode Network– as demonstrated for new antidepressant agents – and ameliorate the cognitive and emotional processing of pain. (Es)ketamine (181, 182) and lamotrigine (177) have shown promising antipsychotic properties, mostly attributed to the NMDAr blockage within specific thalamo-cortical circuits. Accordingly, their properties as potential FND treatments should be thoroughly investigated. Finally, in the frequent cases of comorbid depression or anxiety, therapeutical management should be tailored to the patient’s needs.

## The continuum model and possible variants

Almost two decades after the book (18) on the contemporary approach to Hysteria, Feldman and Yates provided a model for the continuum (98), pointing out where medically recognized diseases, medically unexplained symptoms, and deception would overlap. This model explains the frequent condition where SSD/FND evolve, or rather deteriorate, into Factitious Disorder, while emerging from a recognized disease (Figure 1).

**Figure 3:**
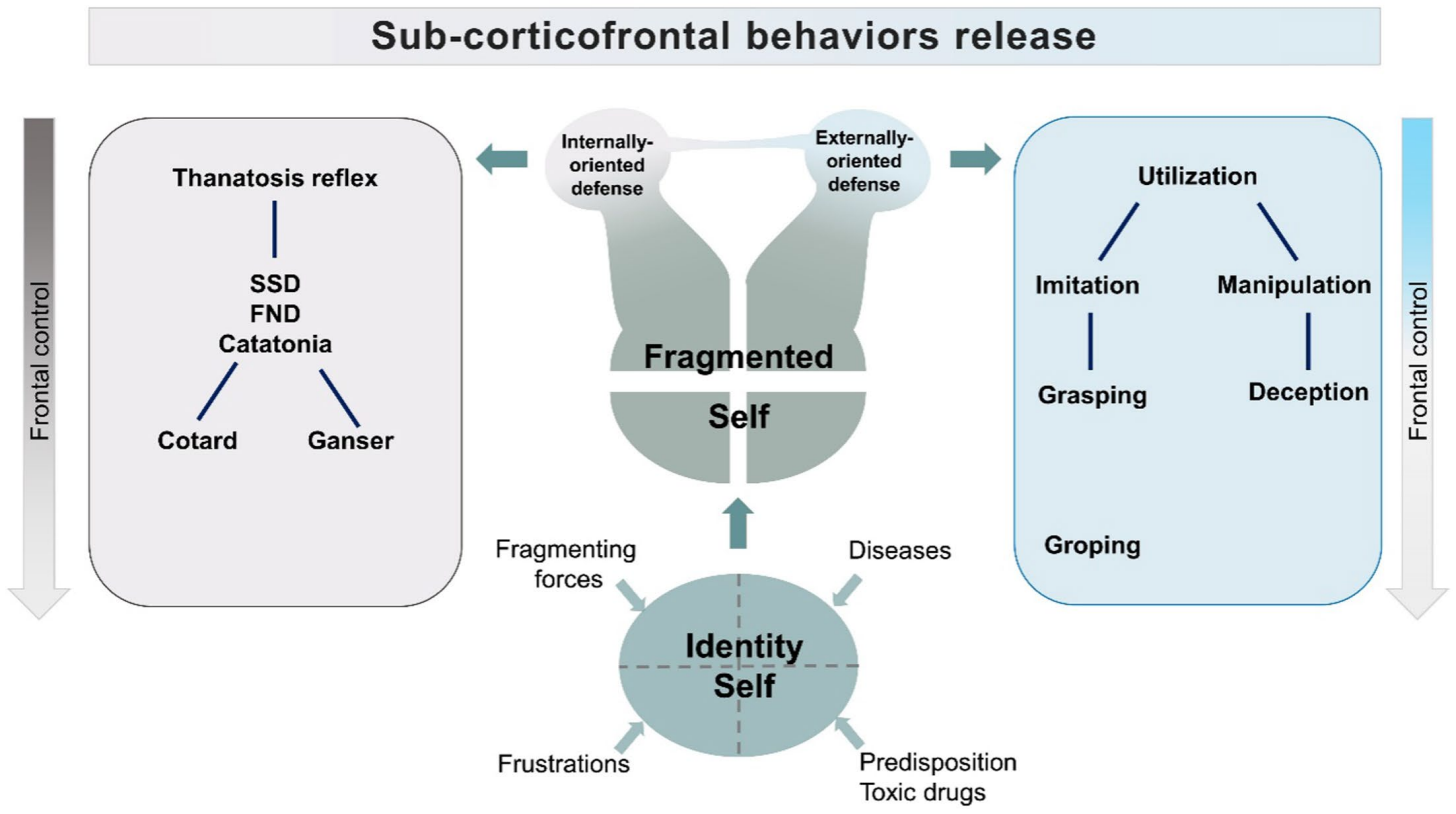
Proposed model. FND, Functional Neurological Disorder; SSD, Somatic Symptom Disorder.

While an undebatable operating instrument for clinical understanding, this model lacks a definition of the background giving origin to the behaviors. From this model, we developed an alternative representation where many variables are inserted and where the equivalence, in terms of severity, and intensity of symptoms, of the three aspects is substituted by a core element consisting of the pulsion to exert control on external and internal forces (Figure 3).

Our model centers on the necessity to exert control, interpreted as a defense against a perceived fragmentation of the identity, i.e., because of an offense to a narcissistic idealization of the self in psychopathological conditions or because of shrinking of higher-order mental activities in neurodegenerative conditions. The attempt to regain control, sometimes accompanied by weakening frontal control networks (30, 178, 183), will produce aberrant behaviors, inclined toward passive or active responses (Figure 3).

The model assumes that the dysfunction of frontal/fronto-parietal control systems will disinhibit activities normally inhibited by Frontal lobes. These activities provide elementary coping patterns, like stimulus bound behaviors, which range from imitation and utilization to the lowest level of grasping and groping. The attempt to regain control of fragmenting forces would generate adaptive elementary coping strategies. Dependent on the extent of frontal lobe dysfunction, the resulting behaviors would include the utilization behaviors, which could be graded in elementary, low-level forms of object manipulation, to higher forms, in which manipulation is addressed to social relations and includes, as a consequence, deception (184, 185). The frontal lobe disinhibited requests for compensation-restitution will induce behaviors reaching levels that will be defined by observers as antisocial, or disruptive. This hypothesis is supported by studies showing that antisocial behavior is linked to dysfunctions of Dorsolateral Prefrontal Cortex, which is the part of the brain activated by social judgement and ethical tasks (186–191). Ethological studies on deception, moreover, do show that this behavior does not need evolute Frontal Lobes to be enacted (192–199). In animals, deception is stratified in different levels, according to the degree of cortical involvement required by the analysis of the environment (200). For instance, freezing represents an automated response to predators. This elementary deception strategy, empowered by subcortical “fear circuits” of the amygdala (201, 202), will be considered in our model as the core reflex subtending the “passive” coping strategy. But it is the higher level of deception, the “tactical deception,” that we consider in the model as the elementary behavior subtending the “active” response, stemming from stimulus-bound utilization behaviors (201, 202). Tactical deception relies on the utilization of normal repertoire contents, to manipulate other individuals of the same species. This level of deception needs a second-order thinking and is common in primates (203). The use of deception among primate species is correlated with the neocortical volumes (204), and appears despite frontal lobes do not reach, in these species, the outstanding, and possibly critical, size proper of the human species.

Therefore, in the model, we indicate two patterns of behavior: a passive coping strategy and an active strategy centered on manipulation. The first pattern is the feigned death reflex activity, which will appear with a range of manifestations including motor arrest, catatonia, Cotard, and Ganser Syndromes (205, 206).

The second pattern is manipulation and deception, which will emerge from utilization/stimulus-bound behaviors.

The two patterns of behavior, rather than randomly overlapping, are represented as stemming from fragmentation of the self, separately or as merging, according to supervening conditions (Figure 3).

**Figure 4:**
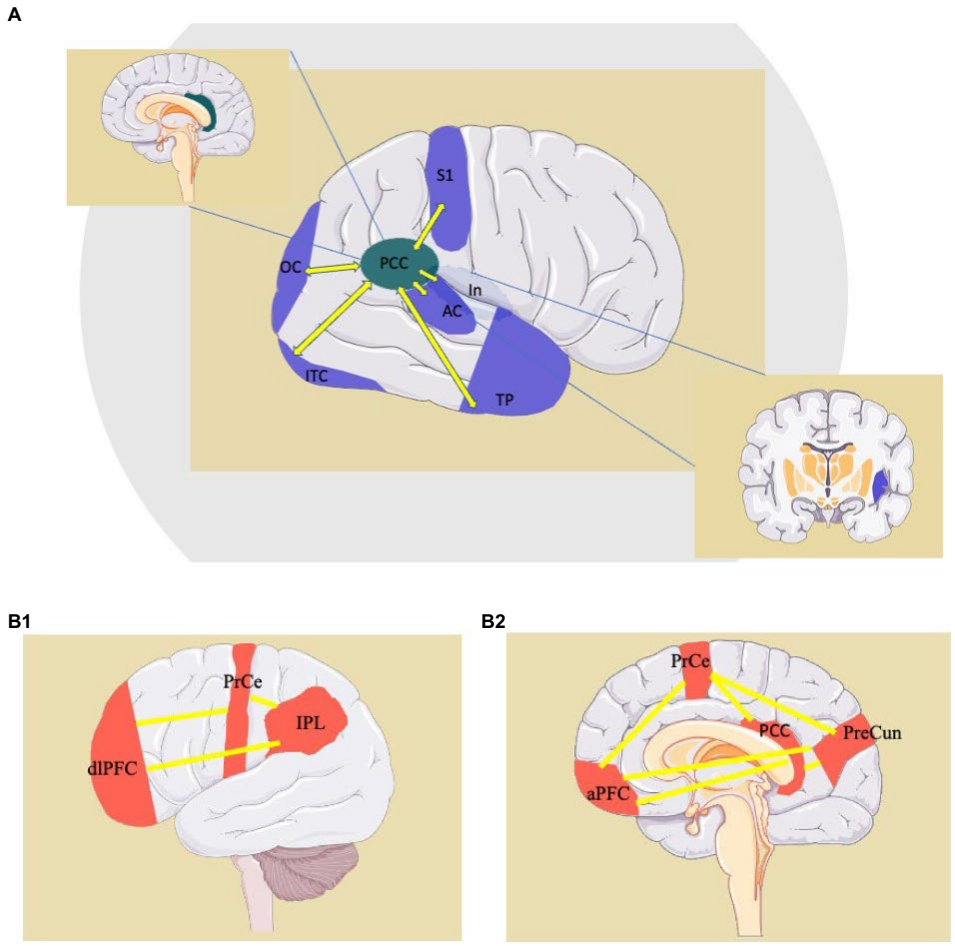
Examples of “small-world” connections of the PCC (Posteriori Cingulate Cortex) **(A)** and representation of the Fronto-Parietal Network, a “Large-World” system involving the PCC **(B1,B2)**. AC: auditory cortex; aPFC: anterior prefrontal cortex; dlPFC: dorsolateral prefrontal cortex; In: Insula; IPL: inferior parietal lobule; ITC: inferior-temporal cortex; OC: occipital cortex; PCC: posterior cingulate cortex; PrCe: precentral gyrus; PreCun: precuneus; S1: primary sensory cortex; TP: temporal pole. http://smart.servier.com.

We suggest that the suppression of “large world” connectivity, and its substitution with “small world” connectivity, observed in neurodegenerative disorders, explain the emergence of elementary behavioral patterns (Figure 4). This substitution is, in our model, represented by the suppression of the, long-distance, “large world” Fronto-Parietal Control Network, and the emergence of short cortico-cortical or cortico-subcortical, “small world” connections. The “Large world/Small World” connection duality was theorized in studies on human brain connectome, and found a prominent space in theories of consciousness (207–216). The connection duality may explain the shift from a modality where the long-range frontoparietal connectivity governs the Posterior Cingulate Cortex to a modality where the lack of inhibition prompts short-range connections and produces behaviors that are resistant to compelling verifications required by consensual reality principles, which are proper of psychotic or dreaming states (212, 213, 217). Based on functional neuroimaging and neurophysiology studies, this model could be applicable to various conditions drawn together by psychotic symptoms and ranging from neurodegenerative disorders to psychedelic states due to the administration of tryptamines. The Posterior Cingulate Cortex is the hub of the Default Mode Network (DMN) (41), a network active during internal narration, suppressed by cognitive tasks (218). The anti-correlation between the activity of the DMN and Task-Positive Networks is necessary to shift focus from internal to external stimuli and plays a pivotal role in the continuous reality check, whose suspension can lead to the onset of psychosis (89, 90). This delicate balance is “peripherally” modulated by the Salience Network, but it heavily depends upon the thalamic pacing. An impairment of thalamo-cortical physiological activity (or ThalamoCortical dysrhythmia, TCD) alters the cortical activity and leads to a decrease of the anti-correlation between task-positive and task-negative systems, an increased propensity to “small-world” functional connections and, eventually, through the generation of random short-range connections motifs, introduces elements of internal narration into external stimuli.

## Conclusion

A growing consensus indicates that FND are heterogeneous conditions that can present with motor or non-motor symptoms. In this context, implementing a multidisciplinary approach can lead to better awareness and management of these conditions. Indeed, neurologists are crucial in examining these patients, find “positive signs,” and providing a broader understanding of the neuroanatomical basis of the physical signs. On the other hand, psychiatrists provide critical neuro-psychoanalytical assessment and holistic management of the patient’s comorbidities, such as anxiety, depression, or personality disorders. This optimal approach requires a reappropriation of cultural competencies by the two disciplines. Unfortunately, in recent years the process has gone in the opposite direction. Experts have often expressed the fear that excessive nosologic parcellation of these conditions may forfeit the social costs and epidemiological relevance of SSD/FND. Furthermore, a perceived reduced relevance of these disorders within the public as produced by again nosologic parcellation is often feared to reduce the allocation of much-needed resources by the National Health systems. Our review provides the conceptual tools to reverse this process.

Our review, therefore, values the wealth of psychopathological approaches, underlining their place in understanding these disorders. By showing the complex history of psychopathological approaches, our review indicates the need to search for additional treatment strategies rather than offering physiotherapy and condescension to symptom presentation.

With an approach integrating psychiatry and neurology competencies, better chances could be offered for responsible insight and growth. Approaches, ranging from behavioral dialectic to cognitive, narrative exposure treatments, tentative inhibition, and pharmacological or interventional techniques, are based on a wealth of validated theories and experimental evidence. These combined strategies prevent the possibility of repeating older assumptions provided at the dawn of studies on the topic (18, 66, 83, 84, 106).

The proposed model mixes psychopathological core mechanisms with functional connectivity changes of cortical networks and biobehavioral expressions of disinhibition and the emergence of subcortical reactive patterns. The model indicates targets to be challenged and points to unresolved methodological issues. We provide the conceptual framework to encourage a multidisciplinary and versatile approach. Instead of a single disease model, our approach aims at bridging the gap between different cultures and disciplines like psychiatry, psychology, neurology, and clinical neurosciences.

## Author contributions

MO and SS: conceptualization. AD, PA, MR, NM, and MO: methodology, writing-original draft preparation. AD, PA, MR, NM, SS, MO, GM, FF, and AT: writing-review and editing. SS and MO: supervision. All authors contributed to the article and approved the submitted version.

## Conflict of interest

The authors declare that the research was conducted in the absence of any commercial or financial relationships that could be construed as a potential conflict of interest.

## Publisher’s note

All claims expressed in this article are solely those of the authors and do not necessarily represent those of their affiliated organizations, or those of the publisher, the editors and the reviewers. Any product that may be evaluated in this article, or claim that may be made by its manufacturer, is not guaranteed or endorsed by the publisher.
